# Legitimation und Kritik der Gemeindepsychiatrie: Begleiterscheinungen und Folgen eines auf Dauer gestellten Modellprojektes

**DOI:** 10.1007/s00115-025-01851-5

**Published:** 2025-06-27

**Authors:** Heiner Fangerau

**Affiliations:** Institut für Geschichte, Theorie und Ethik der Medizin, Medizinische Fakultät, Heinrich-Heine-Universität Düsseldorf, Moorenstr. 5, 40225 Düsseldorf, Deutschland

**Keywords:** Psychiatrie-Enquete, Psychiatriereform, Versorgungsstrukturen, Projektifizierung, Zeitgeschichte, Psychiatry inquiry, Psychiatry reform, Treatment structures, Projectification, Contemporary history

## Abstract

Der Beitrag beschreibt die Entwicklung und Kritik der Gemeindepsychiatrie in Deutschland seit der Psychiatrie-Enquete von 1975 sowie ihre Vorläufer und Referenzpunkte. Während die Enquete grundlegende Reformen wie den Aufbau gemeindenaher Versorgungsstrukturen forderte, blieben konkrete inhaltliche und strukturelle Vorgaben vage. Diese Unschärfe führte dazu, dass die Gemeindepsychiatrie weniger durch zentrale Reformen als durch eine Vielzahl regionaler Modellprojekte geprägt wurde. Mit Blick auf den zeithistorischen Kontext wird diese Projektorientierung als Ausdruck einer Fokusverschiebung auf Selbsthilfe, zivilgesellschaftliche Akteure und Flexibilisierung staatlicher Fürsorge seit den 1980er-Jahren gedeutet. Die Projektifizierung brachte zwar Diversifizierung und Professionalisierung mit sich, führte aber auch zu Unsicherheiten, mangelnder Nachhaltigkeit und Fragmentierung der Versorgung. Die Gemeindepsychiatrie wird so als Spiegel gesellschaftlicher und politischer Wandlungsprozesse verstanden.

## Abstract (en)

This article describes the development and criticism of community psychiatry in Germany since the 1975 Inquiry Commission on Psychiatry as well as its precursors and reference points. While the inquiry called for fundamental reforms such as the establishment of community-based care structures, the concrete content and structural guidelines remained vague. This lack of clarity meant that community psychiatry was characterized less by central reforms than by a large number of regional model projects. With a view to the historical context, this project orientation is interpreted as an expression of a shift in focus since the 1980s towards self-help, civil society actors and the flexibilization of the social state. Although projectification has led to diversification and professionalization, it has also resulted in uncertainty, lack of sustainability and fragmentation of care. Community psychiatry is thus seen as a mirror of social and political change.

## Hintergrund

Als Asmus Finzen 1974 mit einer beispielhaften Bedarfsanalyse „Argumente“ für eine Gemeindepsychiatrie liefern wollte, konstatierte er, dass sein Ansatz nicht darauf abziele, normative Standards zu setzen. Das, so Finzen, sei der Politik vorbehalten [18]. Er adressierte hier mit der Politik einen Handlungs- und Entscheidungsraum, den Psychiater mit ihrer Expertise vor, während und nach Einrichtung der Sachverständigenkommission zur Erstellung des „Berichts über die Lage der Psychiatrie in der Bundesrepublik Deutschland“ aktiv mitzugestalten versuchten [32]. Die Kommission thematisierte in ihrem Abschlussbericht explizit nicht in erster Linie konkrete biomedizinische Maßnahmen, sondern forderte zu einer grundsätzlichen Neuordnung der Versorgung psychisch Kranker auf. Im Blick waren insbesondere der Aufbau bedarfsgerechter gemeindenaher Strukturen für alle Betroffenen, Enthospitalisierung, Entstigmatisierung und die Etablierung ambulanter Dienste [12].

Diese Systemorientierung nahm die Politik in die Pflicht, zog jedoch bei allem Reformgeist einige Probleme und Kritik nach sich. So akkurat, empirisch gesättigt und konkret die Problembeschreibung der Kommission zur Lage der deutschen Psychiatrie war, so vage und unkonkret blieb, was genau mit „gemeindenah“ gemeint war und wie exakt die Umsetzung der Gemeindenähe erfolgen sollte.

Zwar definierte die Sachverständigenkommission eine gemeindenahe Versorgung beispielsweise über die Erreichbarkeit binnen einer Stunde [12]. Was aber die inhaltliche und strukturelle Ausgestaltung anging, konnte sie die Politik – auch aus ihrer Rolle als Expertenkommission heraus – selbstredend nicht festlegen. Seitdem zieht sich ein feiner Riss durch die Idee Gemeindepsychiatrie, den der Psychiater Martin Hambrecht unlängst pointiert mit dem Vergleich auf den Punkt brachte, dass die englische Community Psychiatry durch Inhalte definiert worden sei, während die deutsche Ausdeutung Strukturen, Organisationsformen und Systeme ins Zentrum gerückt habe [22].

Die Wirklichkeit dieser Strukturen erscheint auch 50 Jahre nach der Enquete dysfunktional und optimierbar, wenn man Zustandsbeschreibungen und Forderungen beispielsweise der Diakonie oder anderer Verbände für die 21. Legislaturperiode Glauben schenkt [13]. In diesem Beitrag möchte ich diese Kritik historisieren, indem ich die These verfolge, dass die Kritik an der deutschen Ausprägung der Gemeindepsychiatrie als Ausdruck und Spiegel anderer zeithistorischer Entwicklungen gelesen werden kann, indem sie nämlich Begleiterscheinung und Folge eines auf Dauer gestellten Modellprojektes ist – genauer gesagt, eines Modellprojektes, dessen Charakter darin liegt, dass es wieder Modellprojekte generiert. Zu diesem Zweck werde ich im Folgenden nach einigen einführenden Ausführungen zur Idee und Geschichte der Gemeindepsychiatrie die Praxis der „Projektifizierung“ oder des „Projektemachens“ in Folge der Enquete in den Blick nehmen und zeigen, welche Gründe es für das Projekten eigene „Auf-Zeit-Stellen“ von Modellen für die Gemeindepsychiatrie gab.

## Gemeindepsychiatrie

Mit der Industrialisierung wurde in Deutschland (wie in anderen Ländern) seit der Mitte des 19. Jahrhunderts ein Anstieg der von psychischer Krankheit Betroffenen beobachtet [30]. In der Folge wurde besonders nach den Einigungskriegen das System der Heil- und Pflegeanstalten etabliert und erheblich ausgebaut. Ein treibender Faktor war u. a. das Interesse der Provinzialverbände, möglichst kostengünstig die sogenannten Landarmen versorgen zu können, für die sie im Krankheitsfall aufkommen mussten. Um die eigene Kasse zu entlasten, setzten Provinzialverbände aber nicht nur auf den Ausbau von Anstalten, sondern auch auf private Wohltätigkeit und ziviles Engagement. Sie unterstützten die Gründung psychiatrischer Hilfsvereine, die Betroffenen und ihren Familien Hilfsangebote zur Verfügung stellen sollten [17]. Parallel wurde die Unterbringung von Kranken in Familien im Rahmen der sogenannten Familienpflege etabliert [34]. Nach dem Ersten Weltkrieg standen sowohl Hilfsvereine als auch Familienpflege vor der Herausforderung, dass sie als Relikte aus dem Kaiserreich gesehen wurden [17]. Nun reüssierten Konzepte wie Gustav Kolbs „Offene Fürsorge“ oder Friedrich Wendenburgs „Gelsenkirchener Modell“. Kolb setzte auf die ambulante Fürsorge von in Familien untergebrachten Patienten durch professionelle Anstaltspsychiater, Wendenburg sah die Eingliederung psychisch Kranker in die kommunale Struktur von Fürsorgestellen vor [34]. Es gab also schon vor dem Zweiten Weltkrieg in Deutschland psychiatrische Praktiken einer gemeindenahen Fürsorge außerhalb der Anstalt. Allerdings konnten sich diese Engagements bei allen Erfolgen nie gegen die starke Anstaltspsychiatrie behaupten, die dann auch die Jahre des Nationalsozialismus dominierte und hier ab 1939 zusammen mit den sechs Tötungsanstalten als Infrastruktur für die Patientenmorde fungierte.

Nach dem Ende des Krieges folgte die Bundesrepublik Deutschland dem seit dem Kaiserreich verfolgten Pfad der Psychiatrie als eine anstaltsgebundene Praxis. Es dauerte bis in die 1960er-Jahre, bis Stimmen, die für eine „gemeinschaftsnahe“ Psychiatrie eintraten, Gehör fanden. Diese orientierten sich vor allem an ausländischen Entwicklungen, was auch den Begriff der Gemeinschaftsnähe erklärt [27, 32]. Im englischen Sprachraum wurde nämlich schon seit den späten 1940er/frühen 1950er-Jahren der Versuch unternommen, unter dem Label der „Therapeutic Communities“ die als menschenunwürdig erlebte Verwahrung und Ausgrenzung psychisch kranker Menschen in Anstalten aufzubrechen und an ihre Stelle Integration und Nähe sowie die Gleichstellung mit körperlich Erkrankten zu stellen. Sowohl sozialpolitisch-praktisch (in England angestoßen schon 1945 durch den Bericht des Home Office „The Future Organisation of the Psychiatric Services“) als auch grundsätzlich (in den USA z. B. symbolisiert durch Kennedys Rede „Mental Illness and Mental Retardation“ aus dem Jahr 1963) waren dort Reformen in Gang, die deutsche Beobachter sich auch für die Bundesrepublik erhofften [37].

Das Modell der „Therapeutic Communities“ (maßgeblich verbunden mit der Person des britischen Psychiaters Maxwell Jones) war nicht unumstritten. Hier sollten 20 bis 30 Patienten in einer Abteilung einer Anstalt in einer offenen Gemeinschaft mit den Behandelnden leben. Der dabei auftretende Verlust professioneller Distanz, der bis zur vollständigen Angleichung an die Patientengemeinschaft reichte, wurde von psychiatrischer und antipsychiatrischer Seite scharf kritisiert [20]. In Deutschland fanden diese Modelle Nachahmer Ende der 1960er-Jahre zum Beispiel in Heidelberg. Auch das Sozialistische Patientenkollektiv betrachtete sich selbst als eine „therapeutic community“ [21].

In England wurde seit den 1950er-Jahren zusätzlich nun unter dem Begriff der „Community Psychiatry“ der Zugang verstanden, Patienten in ihrer lokalen Umgebung individuelle Behandlungsangebote zu eröffnen. Freeman und Bennett haben die Idee und Umsetzung für die 1960er-Jahre als undogmatisch, offen für neue Ideen und Befunde sowie pragmatisch und damit in ihrer Zeit erfolgreich bezeichnet [3, 19]. An diese Konzeption schloss sich jetzt der deutsche Begriff der „gemeindenahen Psychiatrie“ an, den Sozialpsychiater rund um die Enquete (als Ersatz für die gemeinschaftsnahe Psychiatrie) stark machten [29]. 1988 folgte dann ein gezielter Begriffswechsel zur „Gemeindepsychiatrie“, womit zum einen auf Kritik an der Enquete bzw. ihrer Umsetzung reagiert wurde und zum anderen der Ansatz der individuellen Versorgung nicht nur am Ort, sondern im gesamten sozialen Netzwerk der Betroffenen noch einmal begrifflich gestärkt werden sollte [1, 32].

Heute erscheint eine „umschreibende Charakterisierung“ dessen, was Gemeindepsychiatrie ist, als der geeignetste Versuch der Definition. Gemeindepsychiatrie umfasst danach einen „bevölkerungsbezogenen Ansatz“, der als System niederschwellige, langfristige, kosteneffektive, erreichbare, lokale Hilfen für Hilfesuchende bereithält, auf Stärken und Bedürfnisse der Betroffenen fokussiert, soziale Netzwerke, Familienstrukturen und andere Beziehungskonstellationen mit einbezieht und dabei evidenzbasiert vorgeht sowie den Geboten einer praktischen Ethik folgt [5].

## Zeitgeschichte, Legitimation und Kritik

Diese umschreibende Definition ist auch das Ergebnis von Auseinandersetzungen über die Umsetzung der angestrebten Psychiatriereform von 1975. Es ging eben nicht nur um die Auflösung der großen Anstalten. Vielmehr sollten nach der Fokussierung auf Strukturen und Logiken des Verwahrens nicht nur rhetorisch, sondern auch praktisch die Menschen und ihre Bedürfnisse in den Mittelpunkt des Handelns rücken. Doch genau an der Praxis schieden sich die Geister. So war der Weg der Enquete mit einigen Paradoxien gepflastert.

Zunächst war er dadurch gekennzeichnet, dass ab den 1960er-Jahren zuerst Anstaltspsychiater auf antipsychiatrische Strömungen reagierten, sie aufnahmen und gleichzeitig ihr Verhältnis zu Reformbewegungen wie der „Sozialpsychiatrie“ ebenso klären mussten wie zu anderen Berufsgruppen oder Laienbewegungen, die zunehmend die Sorge um psychisch Kranke als ihren Gegenstandsbereich betrachteten. Die Anstaltspsychiater fürchteten Deprofessionalisierung und den Verlust von Deutungshoheit [6, 32]. Die „Gemeindenahe Psychiatrie“ formulierten sie als einen praktischen Ansatz, von dem sie auch hofften, dass er als weniger politisierend und linkspolitisch verortet wahrgenommen würde als die „Sozialpsychiatrie“. Gleichwohl waren es gerade Personen, die sich als Sozialpsychiater verstanden, die maßgeblich die Enquete mit vorbereiteten und umsetzten [31].

Ferner bestand konzeptionell die Spannung, dass die Experten der Sachverständigenkommission einerseits die Gleichstellung der psychisch Kranken mit den körperlich Kranken auf ihre Fahnen geschrieben hatten und andererseits mit ihren sozialen Ansätzen die Loslösung vom somatischen Krankheitsmodell propagierten. Praktisch hieß dies etwa, dass die meisten psychiatrischen Akteure der Reform auf den Einsatz der in den 1950er-Jahren aufgekommenen Psychopharmaka setzten, was eine scharfe Trennlinie zu rein sozialen Zugängen oder zur Antipsychiatrie markierte, die biologische Behandlungen ablehnten [20]. Auf institutioneller Ebene wiederum forderten universitätsnahe Psychiater beispielsweise den Ausbau von Ambulanzen, niedergelassene Psychiater nahmen aber genau diese als wirtschaftliche Bedrohung wahr.

Entsprechend waren die Zusammensetzung und Arbeit der Enquetekommission durch vielfältige Kompromisslinien, aber auch Dispute über die hinzuzuziehenden/aufzunehmenden Personen, ihre disziplinäre Ausrichtung, ihre Ziele und Haltungen gekennzeichnet. Zu den umkämpften Feldern gehörte auch die Frage, ob Abteilungen an allgemeinen Krankenhäusern oder den jetzt so bezeichneten „Fachkrankenhäusern“ (den alten Heil- und Pflegeanstalten) die besseren Reformlösungen böten. Heinz Häfner etwa trat für ein zweistufiges System ein, was die Befürworter von Abteilungen wiederum als Abwertung begriffen. Einen weiteren Problembereich, der „Harmonisierungsgespräche“ erforderlich machte, stellte der Einbezug der Psychotherapie und Psychosomatik dar [10, 26, 31, 32, 35].

Diese hier nur summarisch und verkürzt wiedergegebenen Diskussionen waren eingebettet in gesellschaftspolitische Entwicklungen und Diskurse, die den jeweiligen Reformdruck in den Jahren der Enquete und in den folgenden Jahren mitbestimmten. Ein Beispiel bietet hier die Verzahnung von Wissenschaftspolitik mit der Geschichte der Psychiatrie als Argument für die Reform [39]. Im ganzen Reformprozess referenzierten die deutschen Psychiater auf internationale Reformvorbilder, womit sie die deutsche Rückständigkeit zum Argument machten, indem sie implizit und explizit darauf verwiesen, dass der historische wissenschaftliche Anspruch der deutschen Psychiater doch eigentlich ein führender hätte sein sollen [23]. Insbesondere der Ergänzungsband zum Enquetebericht enthält etliches zusammengetragenes Material über gemeindepsychiatrische Erfolge in anderen Ländern [37]. Gleichzeitig versuchten sie nach Jahren des Schweigens, die Patientenmorde im Rahmen der nationalsozialistischen „Euthanasie“-Aktion zu benennen und durch echtes Bemühen um Aufarbeitung Vertrauen zurückzugewinnen. Dies galt sowohl nach außen als auch mit Blick auf die Debatten der 1968er-Generation nach innen. Die kulturelle und politische Bewegung der 1968er hatte der Reform unter anderem auch durch ihre Befassung mit der nationalsozialistischen Vergangenheit einen „Resonanzboden“ eröffnet [23].

Auch wenn Periodisierungen immer strittig sind, so lassen sich doch folgende Trends benennen, vor deren Hintergrund die Idee der Gemeindepsychiatrie am Ende ausgestaltet wurde. Angedacht in den 1960er-Jahren in einer Phase der politischen Liberalisierung und Westernisierung [24] erfolgten Planung und Versuche der Umsetzung „nach dem Boom“ [14] in einer seit der Ölkrise schwieriger gewordenen ökonomischen Situation. Die öffentlichen Kontroversen um soziale Gerechtigkeit, Arbeitsmarkt- oder Familienpolitik nahmen zu, begleitet von einem Wandel normativer Ordnungsvorstellungen, die sich mit Begriffen wie Pluralisierung, Globalisierung, Individualisierung oder Medikalisierung fassen lassen [33].

So lässt sich erklären, dass die Umsetzung von Ideen zur Gemeindepsychiatrie einerseits in Kommunen wie Rheydt systematisch angegangen wurde [36] und andererseits die konzertierte Aktion unterblieb. Sie unterblieb auch, weil die Bundesregierung sich nach der Beauftragung einer Planungsstudie zur Umsetzung der Vorschläge der Enquete vier Jahre Zeit mit einer Stellungnahme ließ. Diese verband sie dann mit der Aussage, dass der Bund nicht zuständig sei und die Länder und Kommunen die Verantwortung für die Reform hin zur Gemeindepsychiatrie übernehmen sollten [32]. Das ohnehin kritische konservativ orientierte *Deutsche Ärzteblatt* kommentierte 1979 trocken zur Psychiatrie-Enquete: „Sie war überflüssig“. Der Chef vom Dienst, Walter Burkart, warnte vielmehr vor einer fehlenden Versorgung von Kranken, wenn die Großanstalten aufgelöst würden [8].

Die Planungsstudie war zu dem Ergebnis gelangt, dass die Vorschläge der Enquete am Ende kostengünstiger seien als bisherige Unterbringungsformen [32]. Auch hier waren die Reaktionen des Ärzteblattes und der Bundesärztekammer bissig. Im Versuch der Delegitimierung wurde die Studie als „wegen der erdrückenden Fülle des Zahlenmaterials, der Schriftgröße und der Sprache nur schwer lesbar“ eingestuft. Ferner wurde lakonisch zur Markierung der vermeintlichen Fehlerhaftigkeit notiert, dass ohne Differenzierung Psychiater und Psychologen nebeneinandergestellt würden. Die Studie stütze sich auf überholte „Bedarfsschätzungen“. Es bestünden am Ende Zweifel, dass die gemeindenahe Versorgung kostengünstiger sei als die existierende Versorgungsform [2].

Es ist Ausdruck des oben angedeuteten gesellschaftlichen Wandels, dass das gleiche Ärzteblatt 22 Jahre später bedauerte, dass die mit der Enquete angestoßene Psychiatriereform „auf halbem Wege stecken geblieben“ sei. Zwar sei „für viele psychisch Kranke“ dank der Reformen eine „‚gemeindenahe‘ Versorgung somit heute möglich“, doch die „flächendeckende sozialpsychiatrische Versorgung“ fehle, u. a. weil viele ausgebaute Strukturen und Akteure noch nicht ineinandergriffen [7].

## Modelle und Projektifizierung

Das 2001 als erreicht Gelobte war gesetzlich gerahmt worden, indem der Bund auf die Enquete reagiert hatte. Unter anderem waren 1976 die Reichsversicherungsordnung an die Bedürfnisse der Ambulantisierung angepasst, 1981 der notorische Halbierungserlass abgeschafft und später teilstationäre Versorgungsmöglichkeiten neu geregelt worden [32]. Zentraler als der Gesetzesrahmen aber war das Auflegen eines Förderprogramms für Modelle, das der Historiker Wilfried Rudloff nicht zu Unrecht als „Modellversuchspolitik“ bezeichnet hat [32]. Am Beginn stand eine ab 1976 laufende Phase diverser Projekte im Rahmen eines Modellverbunds. Die Ergebnisse waren durchwachsen. Deutlich wurde, dass die verwobenen Ziele der Gemeindenähe oft am praktischen Alltag und den lokalen Gegebenheiten scheiterten. Es folgte ein großes von der Bundesregierung aufgelegtes von 1980 bis 1985 laufendes Bundesmodellprogramm „Psychiatrie“, das 14 Modellprojekte fördern sollte. Hier sollten die unterschiedlichen lokalen Dienste (z. B. niedergelassene Ärzte, Beratungsstellen, sozialpsychiatrische Dienste, Wohngruppen, Kliniken etc.) „in ihrem vernetzten Zusammenwirken erprobt werden“ [31]. Es beteiligten sich nur sechs Bundesländer (die unionsgeführten Länder lehnten bis auf das Saarland eine Teilnahme ab, weil sie einen Eingriff in ihre Länderkompetenzen befürchteten), aber weitere legten eigene vergleichbare Projekte auf. Um die Evaluation des Programms und zu ziehende Schlussfolgerungen wurde gerungen. Auch hier setzten am Ende die Psychiater eine mit ihnen besetzte Expertenkommission durch [32].

Die auf Länder- und kommunaler Ebene umgesetzten Projekte unterschieden sich sehr in Umfang und Ausrichtung. Eine Gesamtzahl aller landes- und kommunalen Projekte lässt sich aufgrund der dezentralen und dynamischen Entwicklung nicht beziffern, von mehreren Hundert zu sprechen, ist aber nicht zu hoch gegriffen. Nach der Wiedervereinigung erreichten die Projekte auch die neuen Bundesländer. Einen Einblick in das bis 1991 in Projekten Bewegte und Unerreichte erlaubt die Antwort der damaligen Bundesregierung auf eine große Anfrage der SPD zur „Situation der psychisch Kranken in der Bundesrepublik Deutschland“. Die Antwort schildert eindrücklich den projektbasierten Aufbau gemeindepsychiatrischer Strukturen und länderspezifische Initiativen, konstatiert aber gleichzeitig den teilweise gravierenden Mangel an gemeindenahen Strukturen. Deutlich wird auch der expandierte Einbezug zivilgesellschaftlicher Organisationen in den Ausbau der gemeindenahen Psychiatrie. Explizit gelobt werden u. a. die Reichweiten des 1976 gegründeten „Dachverbands Psychosozialer Hilfsvereinigungen e. V.“ (heute Dachverband Gemeindepsychiatrie) und des 1985 gegründeten „Bundesverbands der Angehörigen psychisch Kranker e. V.“ [12].

Diese Bestandsaufnahme spiegelt in ihrer Schwerpunktsetzung zweierlei Entwicklungen, die sich spätestens mit dem Regierungswechsel 1982 Bahn brachen. Hatte Helmut Kohl in seiner Regierungserklärung davon gesprochen, dass der Staat kollektive Leistungen zurückfahren und persönliche Leistung und Eigeninitiative stärken solle [38], so bilden Projektorientierung und die Verschiebung von Aufgaben in die Selbsthilfe(organisationen) genau diese in der Kritik als „neoliberal“ adressierte Perspektive ab. Dabei werden Selbsthilfe und Laienunterstützung seit den 2000er-Jahren rückblickend zunehmend als Dilemma begriffen. Es wird (international) kritisch angemerkt, dass sich im Zuge der Ökonomisierung die Selbstermächtigung zur Pflicht erhoben habe, womit eine Verlagerung der Verantwortung und der tangiblen wie intangiblen Kosten vom Fürsorgestaat auf die Betroffenen einhergehe [5, 9, 15, 31]. Projekte wiederum stellen kollektive Leistungen auf Zeit.

Boltanski und Chiapello sehen Projekte als elementaren Baustein des Kapitalismus in seiner Ausprägung der auf die 1980er-Jahre folgenden Jahrzehnte. Projektstrukturen stehen hier auch für die Zunahme von Eigenverantwortung und die Flexibilisierung von Arbeitsverhältnissen [4]. Projekte boten in dieser Lesart auf der einen Seite eine Chance, Konzepte mit dem Anspruch auf Optimierung von Lebenssituationen, Sorgestrukturen und Organisationen in der Praxis der Gemeindepsychiatrie auszuprobieren und zu evaluieren. Darüber hinaus ermöglichten sie es, auch Akteure ins Feld zu bringen, die zunächst gar nicht unmittelbar gemeindepsychiatrisch verortet waren, dann aber, wie etwa Projekte zur feministischen Beratung und (Psycho‑)Therapie [28], als Bausteine eines gemeindepsychiatrischen Angebots gesehen werden konnten. Projekte sorgten so für eine Diversifizierung und Professionalisierung von Versorgungsangeboten.

Auf der anderen Seite standen die so geschaffenen Strukturen immer unter Vorbehalt. Das Gleiche galt für die Mitarbeitenden, deren berufliche Perspektive sowohl vom Projektende als auch von einem möglichen Folgeprojekt abhing. Der Projektleiter der Planungsstudie zur Enquete erkannte diese Problematik schon früh und riet 1979, nur Personalausstattungen zu fördern, die auch nach Projektablauf finanzierbar wären [40]. Auch die Deutsche Gesellschaft für Soziale Psychiatrie beurteilte das Projektwesen trotz eigener Beteiligung Anfang der 1980er-Jahre kritisch [31]. Dessen ungeachtet fand das Modellprojektwesen in den folgenden Jahren (bis heute) eine Fortsetzung. Insbesondere durch eine gesetzliche Verankerung von „Modellvorhaben zur Versorgung psychisch kranker Menschen“ in § 64b SGB V (Fünftes Buch Sozialgesetzbuch) sollte eine flexiblere, sektorenübergreifende, patientenorientierte Behandlung ermöglicht und erprobt werden [11].

## Fazit

Die Entwicklung der Gemeindepsychiatrie in Deutschland war das Kernanliegen der Psychiatrie-Enquete. Dabei setzte die Reformpsychiatrie auf bestehende Denk- und Handlungspfade auf. Vor dem Hintergrund der bundesrepublikanischen Entwicklung und zeithistorischen Strömungen fokussierte sie aber mehr als alle früheren Ansätze auf die Bedarfe der Betroffenen und auf Multiprofessionalität. Die Umsetzung gemeindepsychiatrischer Ideen verlief nach dem politischen Erfolg der Enquete dann aber weniger als zentral gesteuerte Reform, sondern vielmehr als eine Folge von Modellprojekten, die wiederum neue Projekte hervorbrachten.

Die Gründe für diese „Projektifizierung“ der Versorgung sind vielfältig. Sie liegen

- in der konzeptionellen Unschärfe bzw. Breite der Idee der Gemeindepsychiatrie, die regional jeweils andere Bedingungen und Zielparameter vorfindet;
- im zunehmenden Netzwerkdenken, das flexible, mobile und variable Konstellationen nahelegte;
- in der Modellgeschichte der Psychiatriereform seit ihren Anfängen, denn bei genauer Betrachtung handelte es sich bei Reformvorschlägen wie der Familienpflege, Offenen Fürsorge oder der Therapeutic Community immer um Modellprojekte;
- in den Spannungen und Kompromissen der Sachverständigenkommission, die unterschiedliche Interessen verschiedenster Akteure vereinen musste, ein Problem, das sich bis heute stellt, was wiederum bedeutet, dass unterschiedliche Umsetzungsideen Projektbedarfe in verschiedenste Richtungen evozierten und weiterhin evozieren;
- in den Kompetenzbereichen des Bundes, der Länder und der Kommunen, die dazu führten, dass der Bund nur auf Projektebene für eine Gemeindepsychiatrie agieren konnte und wollte;
- in der „nach dem Boom“ [14] der Wirtschaftswunderjahre schlechteren Haushaltslage verbunden mit wirtschaftsliberalen politischen Rahmungen, die flexible Projekte attraktiver erscheinen ließen als langfristig mit Kosten belegte Dauerlösungen.

Die „Projektifizierung von allem“ [25] ergriff die Gemeindepsychiatrie also von Anfang an mit voller Wucht. Sie wurde zu einem ihrer konstitutiven Merkmale. Sie durchdrang auch das gemeindepsychiatrische Denken in Managementstrukturen [15]. Die Projektaktivität eröffnete und schuf neue Räume und professionelle Beziehungskonstellationen, dies aber immer unbestimmt, temporär und mit der Bereitschaft, schnell neue Konstellationen zu adressieren. Projekte blieben somit stets Durchgangsstationen [25]. Sie führten zwar zur gewünschten Diversifizierung, dauernden Evaluation und Professionalisierung der Angebote, brachten aber auch Unsicherheiten, mangelnde Nachhaltigkeit und eine fortwährende Fragmentierung der Versorgung mit sich. Damit spiegelt die Gemeindepsychiatrie gesellschaftliche und politische Wandlungsprozesse der letzten 50 Jahre wider, mehr noch, sie ist ein Feld anhand dessen sich zeithistorische Entwicklungen wie durch ein Brennglas im Sinne einer „psychiatrischen Zeitgeschichte“ [16] betrachten lassen.
